# Context Matters: Revisiting the First Step of the ‘Sequence of Prevention’ of Sports Injuries

**DOI:** 10.1007/s40279-018-0953-x

**Published:** 2018-06-28

**Authors:** Caroline Bolling, Willem van Mechelen, H. Roeline Pasman, Evert Verhagen

**Affiliations:** 1 Amsterdam Collaboration for Health and Safety in Sports, Department of Public and Occupational Health, Amsterdam Public Health Research Institute, VU University Medical Center, Van der Boechorststraat 7, 1081 BT Amsterdam, The Netherlands; 2 UCT/MRC Research Unit for Exercise Science and Sports Medicine (ESSM), Department of Human Biology, Faculty of Health Sciences, University of Cape Town, Cape Town, South Africa; 3 School of Public Health, Physiotherapy and Population Sciences, University College Dublin, Dublin, Ireland; 4 School of Human Movement and Nutrition Sciences, Faculty of Health and Behavioural Sciences, University of Queensland, Brisbane, QLD Australia

## Abstract

It is possible to prevent sports injuries. Unfortunately, the demonstrated efficacy and effectiveness of injury prevention approaches are not translated into lasting real-world effects. Contemporary views in sports medicine and injury prevention suggest that sports injuries are ‘complex’ phenomena. If the problem we aim to prevent is complex, then the first step in the ‘sequence of prevention’ that defines the ‘injury problem’ already needs to have considered this. The purpose of this paper is to revisit the first step of the ‘sequence of prevention’, and to explore new perspectives that acknowledge the complexity of the sports injury problem. First, this paper provides a retrospective of the ‘sequence of prevention’, acknowledging contemporary views on sports injuries and their prevention. Thereafter, from the perspective of the socioecological model, we demonstrate the need for taking into account the complex nature of sports injuries in the first step. Finally, we propose an alternative approach to explore and understand injury context through qualitative research methods. A better understanding of the injury problem in context will guide more context-sensitive studies, thus providing a new perspective for sports injury prevention research.

## Key Points

**Table:** 

Contemporary views in sports medicine highlight the complexity of sports injuries and their prevention.
The widely used ‘sequence of prevention’ needs to take the complexity into account, starting with the first step ‘describing sports injury problem’.
Introducing qualitative research approaches as a means to include complexity of the sport injuries in the problem description provides ways to more comprehensively understand the sports injury context.

## Introduction

Since the ‘sequence of prevention’ of sports injuries [1] was published, many studies have shown that under ideal (i.e. controlled) and pragmatic (i.e. real-world) conditions, it is possible to reduce injury incidence, injury severity and the costs associated with sports-related injuries [2]. The outcome of controlled trials is referred to as ‘efficacy’, whereas the outcome of pragmatic trials is defined as ‘effectiveness’. Unfortunately, the demonstrated efficacy and effectiveness results are not translated into a lasting meaningful effect in the real world [3–6]. Consequently, implementation has become a major question, i.e. ‘how to translate evidence into practice?’.

To overcome this implementation gap in our field, Finch has proposed the “Translating Research into Injury Prevention Practice” (TRIPP) framework [4]. TRIPP adds two additional steps to the ‘sequence of prevention’: (1) the need for understanding the implementation context (personal, environmental, societal and sports delivery factors); and (2) the evaluation of the implementation process of preventive measures [4]. The need for an evaluation of the implementation process highlights our limited understanding of the sports context as a potential driver of preventive behaviour [7]. Thus, knowledge is required about the setting, the culture, and the infrastructure related to sports injuries, which could be so-called contextual determinants of the injury prevention process.

In the Oxford Dictionary, ‘context’ is defined as “the interrelated conditions in which something exists or occurs” [8]. Drawing a parallel with sports injury, this implies that for the same injury, the ‘injury problem’ can differ, based on differences in context, e.g. between a circus artist, an elite athlete, or a professional dancer. A dancer can deny or not report an ankle sprain and still perform because she is afraid to lose her position as a soloist, whereas a basketball player, with the same type of ankle sprain, will normally be out of training for a week or more, but will probably continue playing if the injury happens before an important final game in the play-offs. However, if we look at the injury from just a biomedical perspective, it is the same injury, with equal tissue damage and clinical prognosis. Yet, when the context is considered, these injuries present different problems, which will consequently require different preventive (and curative) solutions. The development of such solutions should then logically be based on the athlete and his or her context prior to the implementation of any solution, and should take into consideration the demands, needs, possibilities and motivation of the athlete.

These contextual aspects of sports injury should already be described and analysed as part of the problem description, i.e. in the first step of the ‘sequence of prevention’. Consequently, this paper revisits the first step of the ‘sequence of prevention’, introducing context as part of the sports injury problem definition, and proposes a new approach to explore the context in which injuries occur.

First, we explain how the importance of context has led to the introduction of complexity in sports injury prevention. Thereafter, this conceptual paper provides a retrospective on the ‘sequence of prevention’ in light of the complexity paradigm. From there, we demonstrate the need to take into account context complexity at the beginning of the injury prevention process. We conclude this review by proposing and emphasizing the need to consider context-specific research questions, and highlighting the need for a greater emphasis on qualitative methods being used in sport injury prevention research.

## Contemporary Sports Medicine is Complex

As already mentioned, contemporary views in sports medicine support the notion that sports injuries are ‘complex’ and propose an ecological and dynamic systems approach towards injury prevention interventions. Bittencourt et al. [9] proposed a framework that challenges the current reductionist approach to sports injury aetiology, presenting ‘complexity’ as an alternative paradigm to understand the occurrence of sports injury. Following the same reasoning, Bekker and Clark [10] suggested analysing injury through the lenses of complexity, acknowledging the importance of understanding the influence of context in sports injury research. Finally, Hulme and Finch [11] advocated the need to introduce a complementary systems paradigm for a better understanding of the process of preventing sports injuries.

If the sports injury problem is acknowledged to be complex throughout the steps of the ‘sequence of prevention’, as the three views outlined above propose, then the first step that defines the ‘injury problem’ needs to have already considered this complexity and the context of sports injury before moving to the next steps. After all, if one starts with no or limited knowledge about the context of a sports injury, this will lead to the development of context-free preventive solutions.

As an analogy, let us say we are using our efficacy knowledge to put our resources into building a Formula 1 car. The Formula 1 car performs perfectly under controlled conditions, such as a pristine Formula 1 track. However, if our Formula 1 car then had to be used in everyday conditions, we would realize that we would need to drive it on country backroads. If we had known the conditions it had to drive on at the outset, we would have built a 4 × 4 instead, i.e. a much slower alternative, but better aligned to the context in which it needs to operate. Now to make our efforts worthwhile, within our current research paradigm we try to modify the road for it to fit our Formula 1 car, rather than going back to the design table to create a car that actually fits the context. This is also exactly what happens in sports injury prevention research.

Efficacious interventions are developed and tested under controlled conditions, after which we attempt to change the users’ behaviour to adopt our ‘ideal’ intervention. Verhagen [12] has argued that this is not the right approach and stated that injury prevention efforts need to be built around athlete behaviours to be effective. Injury prevention should focus on ‘what works for whom, when, where and why’ [10]. Consequently, there is a need to know and understand more about the behavioural aspects related to injury occurrence.

## Revisiting the Steps of the ‘Sequence of Prevention’

### Step 1: Describe the Sports Injury Problem

**Fig. 1:**
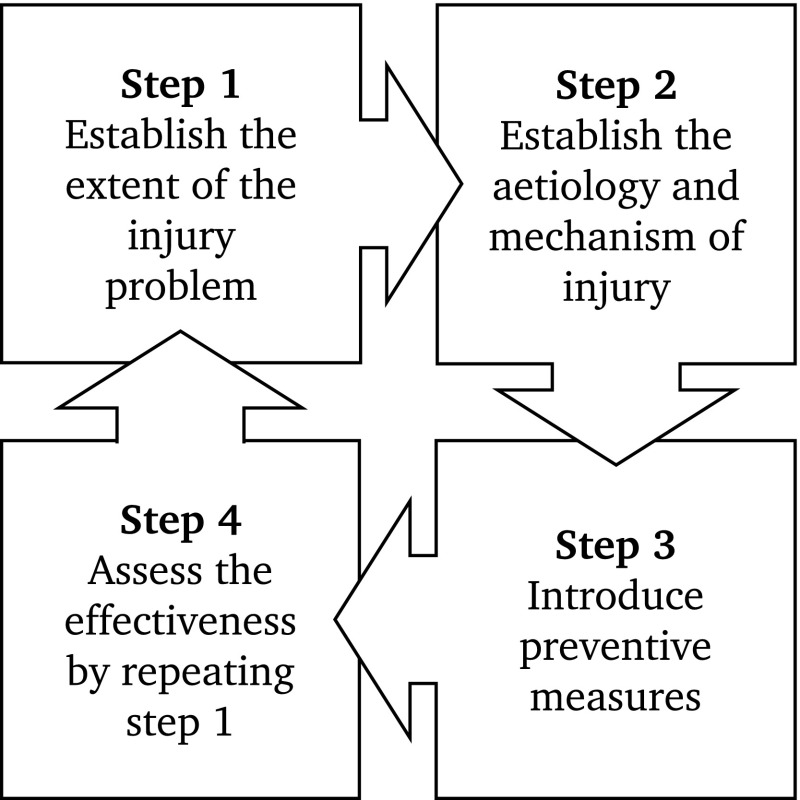
The ‘sequence of prevention’ of sports injuries (adapted from van Mechelen [1], with permission)

In the first step, the sports injury problem is described by the magnitude of the problem and its severity (Fig. 1). The problem ‘injury’ is typically measured by epidemiological measures and quantified in epidemiological studies. Prevalence, incidence, severity, injury profiles, time loss, and costs have all been well-described in a wide variety of sports, and stratified by age, sex, participation level, sports, experience, etc. [2].

### Step 2: Describe Injury Aetiology

Historically, based on traditional biomedical and pathophysiological aetiology, the cause–effect paradigm has been applied widely in sports injury research, focusing on finding the independent effect of a risk factor on injury outcome. Knowledge about risk factors is mainly established through biomechanical, biomedical and epidemiological research [6]. Many biomechanical and neuromuscular factors have been identified as risk factors for the occurrence of lower-limb injuries in many sports [13]. However, most risk factor studies assume sequential linearity to explain the occurrence of sports injury and deal mainly with downstream risk factors, which are proximate to the injury event and are individual-related [14]. On the other hand, upstream risk factors are factors largely outside the control of the individual, for instance psychosocial factors, which are less targeted in sports injury research [15–17].

The multifactorial and dynamic nature of injuries has been recognized by distinguishing the intrinsic and extrinsic risk factors that lead to injury, yet in a sequential linear way [18]. More recently, Bittencourt et al. [9] introduced the concept of a web of determinants (i.e. risk factors), which implies a complex and dynamic systems approach, in a multiple-levels network. Following this web of determinants model, each context will present multiple pathways that may lead to comparable injury outcomes.

### Step 3: Introduce Preventive Measures

The third step is to introduce measures that are likely to reduce the future risk and/or severity of sports injuries. These measures should be based on the risk factors and the mechanism(s) identified in the second step. Consequently, the following interventions should target well-described, modifiable risk factors. In this third step, emphasis is typically placed on controlling or changing the environment (i.e. external risk factors) and/or on modifying intrinsic person-related risk factors. Because behaviour is supposed to modify risk factors and injury mechanisms, behaviour is seen as an important component of the injury prevention intervention [6]. Adopting preventive interventions aimed at reducing sports injury risk can be regarded as health behaviour. However, from a public health perspective, the study of any health behaviour in isolation from the broader social and environmental context is incomplete and will lead to disappointing results when experiments targeting behaviour change are transferred into the ‘real world’ [19]. For example, interventions to prevent overweight or to increase physical activity are more successful when multilevel, contextual, socioecological factors (e.g. cultural background and environmental changes) are understood and considered when translated into practice [20].

Verhagen et al. [6] have already pointed out the need to address behaviour and to identify determinants of sports behaviour when attempting to prevent sports injuries. McGlashan and Finch [17] reviewed and analysed the use of behavioural and social sciences theories and models in research, and found that most sports injury prevention studies applied individual-level (intrapersonal/interpersonal) theories. Their review showed that organizational- and community-level theories have been used rarely. The latter types of theories tend to assume that context is inherent to preventive behaviour and consider more than the individual. More recently, Vriend et al. [21] reviewed intervention strategies for sports injury prevention and concluded that most of the interventions had targeted the individual. Based on the limited understanding of behaviour as a key determinant of effective injury prevention applied in sports injury research, McGlashan and Finch [17] have recommended that future sports injury prevention studies should consider the complexity of sports behaviours.

### Step 4: Evaluation of Introduced Measures

Given that interventions have been developed, the fourth step aims to ensure that measures actually ‘work’. Many interventions have been tested in a controlled and relatively context-free environment and have shown to be able to prevent injuries. Subsequent studies have studied proven efficacious interventions in more practical (i.e. less controlled) environments and have evaluated effectiveness and cost effectiveness [2]. However, research has also demonstrated that many other factors affect intervention effectiveness, including factors such as intervention adherence, attitudes and beliefs [17]. This notion has led to the development of the sequence of steps in the TRIPP framework [4], which targets the implementation process of efficacious interventions.

### Additional Steps to Support Implementation

The implementation process has also been evaluated to obtain a better understanding of the limited effect of interventions in effectiveness studies [22]. To this end, the RE-AIM framework [23] has been applied to injury prevention interventions to evaluate reach, effectiveness, adoption, implementation and maintenance of such interventions. This RE-AIM framework provides ‘contextual’ parameters to evaluate the intervention and its implementation.

A systematic review evaluating 52 published injury prevention trials using the RE-AIM framework identified that major gaps in the implementation of preventive measures can be identified in the adoption and maintenance of interventions [24]. These two aspects of the implementation process relate to the behaviours of end users, who need to adopt the measures and support their maintenance. The review stipulated the importance of the recognition of multiple stakeholders within a sports system who are relevant for effective implementation of intervention measures, including players, coaches, staff and administrators.

A review of the use of the RE-AIM framework in public health research [25] also indicated an increased awareness of the importance of context and provides support for asking questions such as ‘which complex intervention for what type of complex patients, delivered by what type of staff, will be most cost-effective, under which conditions, and for what outcomes?”. Thus, contextual factors should be taken into consideration earlier in the process of the development of a sports injury prevention intervention and not just in the implementation phase.

## You Cannot Expect Good Wine from Bad Grapes

In a recent editorial, Bekker and Clark [10] questioned the ‘sequence of prevention’ if the only question is ‘does the intervention work or not?’. They argued that the ‘sequence of prevention’ is simplistic, considering the complexity of the sports injury. However, the sequence provides, just as any framework, merely a way to operationalize the research process (Fig. 1). In a production process, the feedstock, i.e. the raw material, will determine the final product. When thinking about the four steps of the ‘sequence of prevention’, it is important to recognize that the feedstock (i.e. the first step) is the problem description and is highly important in regard to the processes and outcomes of subsequent steps.

**Fig. 2:**
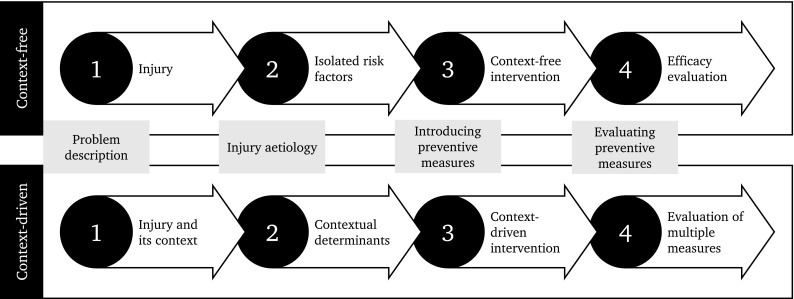
In the past 25 years, the ‘sequence of prevention’ of sports injuries has been mostly applied to produce context-free evidence, i.e. describing the problem and following the steps in controlled environments that did not consider the context as part of the problem from the outset. Contemporary views demand the ‘sequence of prevention’ to be context-driven, which will provide a more comprehensive view of the injury problem and effective solutions

Over the years, the content of the steps of the ‘sequence of prevention’ has been revisited and new context-driven perspectives have been added. Within the second step, new insights have been proposed which consider aetiology as consisting of a ‘web of determinants’ that should be understood from the perspective of a ‘complex systems approach’ [9–11]. In the third step, some studies have shown the importance of incorporating socioecological theory to understand behaviour [17, 26, 27]. In the fourth step, and subsequent implementation steps, recent studies have highlighted the importance of taking contextual determinants into account when the success of an intervention and its implementation are evaluated [24]. However, the first step, in which we describe the problem, seems to still be performed with a context-free narrow focus on the injury (Fig. 2).

## A Fresh Start for the ‘Sequence of Prevention’

**Fig. 3:**
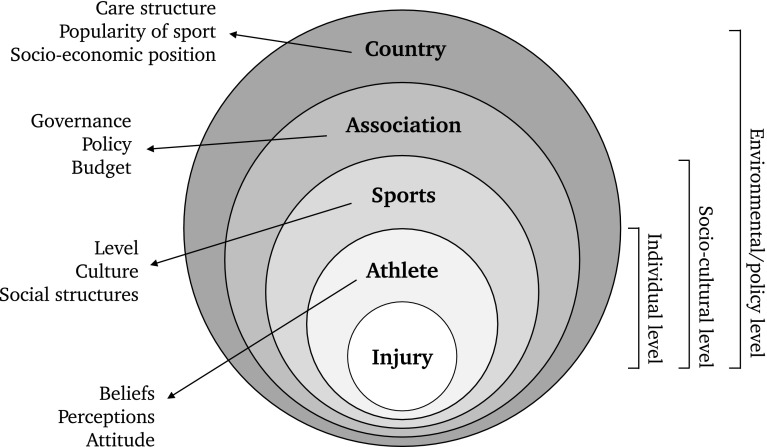
A socioecological view of sports injuries that includes context at multiple levels, i.e. individual, sociocultural and environmental

The injury will involve an athlete who can be characterized by many individual features as well as multiple extra-individual factors that are associated with injury risk, e.g. the level of performance in a particular sport, which has a specific culture, is regulated by a specific association and takes place in a particular socioeconomic class in a specific country (Fig. 3).

A recommended approach would be to consider the injured athlete being in the centre of a socioecological model, an approach already presented for medical interventions and health policies [28]. Bronfenbrenner and Ceci [29] postulated that to understand an individual’s development and behaviour, the entire ecological system in which this individual acts needs to be taken into account. The recent paper by Ekstrand et al. is, for instance, a good example of how coach leadership style, as part of an ecological system, is related to soccer injuries [30]. Some authors have already presented a socioecological model as a framework for analysing the preventive behaviours in sports injury causation [17, 26, 27, 31]. If the injured athlete is placed at centre stage in the viewpoint, the socioecological model can help to understand the dynamic interrelations between, among others, physical, biological, ecological, technical, economic and social aspects.

## Exploring the Context

### New Questions

There is a need to solve problems in sports medicine practice, and, in order to do that, the injury problem needs to be addressed in context. It is necessary to understand the athlete’s context and how this context affects injury, how the athlete perceives injury, and how the athlete deals with prevention. Instead of asking whether an intervention works for a specific problem, new questions should ask how context impacts a problem [32].

Public health has been challenged by similar questions for many years (and still is). For example, ‘condom use’ is a simple preventive measure that, despite its proven efficacy, is not always current practice. Some studies have tried to understand why teenagers refrain from using a condom even though they acknowledge the risk of disease and unintended pregnancies [33]. The answers were found by qualitative research methods, showing the relevance of contextual factors that play an important role in behaviour, in this case partner influence and social acceptance, for example [33, 34]. To further illustrate challenges in the context of sport injury prevention, Bahr et al. [3] conducted the Nordic Hamstring Survey. It was found that teams do not adopt and implement an exercise programme with a well-documented effect on both injury and re-injury risk, but continue to use exercises with no or limited supporting evidence [3]. Such outcomes underline the necessity of new research methods to better understand the contextual determinants of a specific prevention process.

### New Research Method Approaches Needed

New context-specific questions on the ‘how’ and ‘why’ need to be posed in sports injury prevention research in order to provide better understanding of the injury in its context [35]. Given the complex nature of sports injuries, there is a need for a greater depth of understanding of the problem than the current knowledge attained mostly from quantitative methodologies. Embracing complexity requires changes in the way that we build evidence, for instance by incorporating qualitative methods in our studies. This idea to incorporate qualitative methods is part of building evidence about complex phenomenon [10, 36]. Qualitative methods can address gaps in our understanding of a process and can provide a contextual perspective on the problem, yielding insights not previously studied [37]. Qualitative methods are becoming increasingly prevalent in medical research, however qualitative research has been limited in the sport injury prevention area [38–43]. Qualitative methods provide ways for researchers to explore and explain contexts, enabling a more comprehensive understanding of many aspects of health [44]. The context shapes evidence and qualitative research can highlight aspects that would usually fall outside the purview of traditional evidence [45].

Many pieces of the puzzle are missing because of the limited understanding of the complexity of the sport context in which an injury occurs. Prior to measurement and quantification of the problem, qualitative methods can explore this context and describe in-depth insights, based on the perspectives of athletes, coaches and health providers [38, 41–43]. Their view might provide a better understanding of injury occurrence and a more comprehensive way to describe the ‘sports injury’ problem.

Qualitative research is more than just ‘another method’. It requires a different approach to the problem, instead of the positivistic approach that attempts to find a unique truth that can be generalized, i.e. the most common approach in quantitative research [46]. Qualitative research accepts a more naturalistic approach, recognizing multiple realities and seeking to understand and interpret relationships between different realities. The different perspectives that surround the sports injury arise from different beliefs and assumptions, which shape the way that a coach, athlete or health provider will behave.

## Conclusions

After the first step of the ‘sequence of prevention’, armed with a broad view of the injury problem and a deeper understanding of the context, the next steps will evolve further into context-sensitive research evidence, giving better grounds for injury prevention research. To explore sports injury questions, researchers need to understand and explore the context in which injuries occur. By applying qualitative methods in sport injury prevention research, we will be able to gain an in-depth understanding of the context in which injuries occur. Instead of translating science to practice, we need to take context into account in order to speak a common language. Once this has been done, tailored interventions can be designed, implemented and tested in the real world, rather than trying to transfer customised programmes based on proven efficacious interventions into the real world with limited effectiveness.
